# Biomedical Promise of Sustainable Microwave-Engineered Symmetric Curcumin Derivatives

**DOI:** 10.3390/pharmaceutics16020205

**Published:** 2024-01-31

**Authors:** Cristina Doina Niţu, Maria Mernea, Raluca Ioana Vlasceanu, Bianca Voicu-Balasea, Madalina Andreea Badea, Florentina Monica Raduly, Valentin Rădiţoiu, Alina Rădiţoiu, Speranta Avram, Dan F. Mihailescu, Ionela C. Voinea, Miruna Silvia Stan

**Affiliations:** 1 Department of Anatomy, Animal Physiology and Biophysics, Faculty of Biology, University of Bucharest, 91–95 Splaiul Independenţei, 050095 Bucharest, Romania; cristina.nitu@iob.ro (C.D.N.); maria.mernea@bio.unibuc.ro (M.M.); speranta.avram@bio.unibuc.ro (S.A.); dan.mihailescu@bio.unibuc.ro (D.F.M.); 2 Institute of Oncology “Prof. Dr. Al. Trestioreanu”, 252 Sos. Fundeni, 022328 Bucharest, Romania; 3 Department of Biochemistry and Molecular Biology, Faculty of Biology, University of Bucharest, 91–95 Splaiul Independentei, 050095 Bucharest, Romania; bizon.raluca_ioana@s.bio.unibuc.ro (R.I.V.); voicu.balasea-bianca@s.bio.unibuc.ro (B.V.-B.); madalina-andreea.badea@bio.unibuc.ro (M.A.B.); miruna.stan@bio.unibuc.ro (M.S.S.); 4 Interdisciplinary Center of Research and Development in Dentistry (CICDS), Faculty of Dental Medicine, “Carol Davila” University of Medicine and Pharmacy, 020021 Bucharest, Romania; 5 Laboratory of Functional Dyes and Related Materials, National Research and Development Institute for Chemistry and Petrochemistry—ICECHIM, 202 Splaiul Independentei, 6th District, 060021 Bucharest, Romania; monica.raduly@icechim.ro (F.M.R.); vraditoiu@icechim.ro (V.R.); coloranti@icechim.ro (A.R.)

**Keywords:** curcumin derivatives, bisdemethoxycurcumin, cervix cancer, anti-tumor effects

## Abstract

Curcumin is a polyphenol of the *Curcuma longa* plant, which can be used for various medicinal purposes, such as inflammation and cancer treatment. In this context, two symmetric curcumin derivatives (D1—(1E,6E)-1,7-bis(4-acetamidophenyl)hepta-1,6-diene-3,5-dione and D2—p,p-dihydroxy di-cinnamoyl methane) were obtained by the microwave-based method and evaluated for their antitumoral effect on human cervix cancer in comparison with toxicity on non-tumoral cells, taking into account that they were predicted to act as apoptosis agonists or anti-inflammatory agents. The HeLa cell line was incubated for 24 and 72 h with a concentration of 50 μg/mL of derivatives that killed almost half of the cells compared to the control. In contrast, these compounds did not alter the viability of MRC-5 non-tumoral lung fibroblasts until 72 h of incubation. The nitric oxide level released by HeLa cells was higher compared to MRC-5 fibroblasts after the incubation with 100 μg/mL. Both derivatives induced the decrease of catalase activity and glutathione levels in cancer cells without targeting the same effect in non-tumoral cells. Furthermore, the Western blot showed an increased protein expression of HSP70 and a decreased expression of HSP60 and MCM2 in cells incubated with D2 compared to control cells. We noticed differences regarding the intensity of cell death between the tested derivatives, suggesting that the modified structure after synthesis can modulate their function, the most prominent effect being observed for sample D2. In conclusion, the outcomes of our in vitro study revealed that these microwave-engineered curcumin derivatives targeted tumor cells, much more specifically, inducing their death.

## 1. Introduction

*Curcuma longa*, the turmeric plant, contains several significant components with anti-tumor, antioxidant, and anti-inflammatory activities, which include curcumin (diferuloylmethane) as the most important and main active compound, along with desmethoxycurcumin (curcumin II) and bisdemethoxycurcumin (curcumin III) [1]. It was shown that curcumin could induce the activity or expression of phase II carcinogen detoxifying enzymes to provide preventive effects against cancer and can inhibit carcinogen bioactivities via cytochrome P450 enzyme suppression [2]. Different proliferative signaling pathways, such as NFκB, AP1, Sonic Hedgehog, TGF beta, JAK-STAT, MAPK, and Wnt-β-catenin, can be suppressed by curcumin, inducing apoptosis [3,4].

The inhibitory effect exerted by curcumin on NF-κB is important because the activation of this pathway is higher in tumor cells compared to normal ones, being involved in the development of carcinogenesis and metastasis. Thus, curcumin treatment tends to prevent phosphorylation and degradation of IkB (NF-κB inhibitor), and NF-κB tends to maintain its binding to the inhibitor. The inactivated NF-κB/IκB complex is kept in the cytoplasm and is not able to enter the nucleus. As a result, the carcinogenesis-related expression of genetic products of NF-κB, including cyclin D1, COX-2, and Bcl-2, is down-regulated by curcumin in various tumor cells [5].

Although curcumin has been taken into consideration for a wide range of biological activities, there are several concerns about using this compound as a therapeutic agent [6]. The poor aqueous solubility, rapid metabolism, and relatively low bioavailability have been highlighted as the major problems. These issues have made researchers find alternatives for improved curcumin delivery systems and new synthetic curcumin derivatives to overcome the limitations and obtain higher efficacy with minimum toxicity [7]. Frequent changes made in curcumin structure included modification of aryl sidechain, di-keto function, double bond, active methylene functionality, the addition of curcumin metal complexes, and curcumin structural analogs [8]. 

The results showed that curcumin derivatives have better stability in blood, higher water solubility, and significant bioavailability and bio-distribution than curcumin. Also, they had a five times higher efficiency than curcumin for inducing apoptosis in breast cancer [9]. However, there is still controversy regarding the ability of curcumin to exert antiproliferative effects against cancer cells, while the non-tumoral cells remain safe with little or no side effects. Recently, curcumin has been shown to have differentiated effects on normal cells, maybe due to the higher cellular absorption in cancer cells than in normal ones [10]. Taking into account these challenges, the main purpose of our study was the in vitro analysis of the antitumor properties of some curcumin derivatives obtained by green synthesis against cervical cancer cells, compared to the possible toxic effects on non-tumoral cells in order to highlight the specificity of curcumin’s action on malignant cells.

## 2. Materials and Methods

### 2.1. Synthesis and Characterization of Curcumin Derivatives

The synthesis method of the two curcumin derivatives with β-diketone structure has already been described in our previous work [11]. Curcumin derivatives structures were confirmed by ^1^H-NMR and ^13^C-NMR spectra on a BRUKER AVANCE 400 MHz spectrometer (Bruker, Billerica, MA, USA) at 25 °C, using CDCl_3_ and tetramethylsilane solutions as the internal reference. These results were presented in our previous work [11]. 

UV-VIS spectra of curcumin derivatives and standard solvated in methanol (0.0625 mg/mL) were measured using a Specord 200 Plus spectrophotometer (Analytik Jena, Jena, Germany). Fluorescence emission spectra of samples (0.1 mg/mL) were measured using a FlexStation 3 Multi-Mode Microplate Reader (Molecular Devices, San Jose, CA, USA), considering an excitation wavelength of 435 nm. 

Dry samples of derivatives and standards were analyzed by Fourier-transformed infrared (FTIR) spectroscopy using a Bruker Tensor 27 spectrometer (Bruker, Ettlingen, Germany). Measurements were performed in attenuated total reflection (ATR) configuration. The samples were directly placed on the ATR crystal without additional processing and were pressed down to ensure optimal contact with the crystal. The spectra were recorded with a resolution of 4 cm^−1^, each spectrum representing the mediation over spectra acquired during 1 min. The absorption maxima of each spectrum were identified with OPUS software version 7.2 (Bruker Optik GmbH, Ettlingen, Germany). 

### 2.2. Computational Analysis of Curcumin Derivatives

The compounds were drawn, and their SMILES structures were generated using Marvin JS version 23.12.0, (https://marvinjs-demo.chemaxon.com/latest/demo.html, accessed on 5 September 2023). Using their SMILES structures, we performed various predictions as follows.

#### 2.2.1. Prediction of Drug-Likeness Profile and Bioavailability

The physicochemical properties of compounds were calculated using the SwissADME server [12]. The platform also predicts the drug-likeness profile of compounds by applying the rules of Lipinski [13], Ghose [14], Veber [15], Egan [16], and Muegge [17]. The oral bioavailability radars, the bioavailability scores, pan assay interference structures (PAINS), structural alerts, and the lead-likeness of compounds were also calculated by the SwissADME server [12].

#### 2.2.2. Pharmacokinetic and Toxicity Profiles of Compounds

The absorption, distribution, metabolization, and excretion (ADME) profiles of compounds were calculated using the SwissADME [12] and pkCSM web server [18]. The toxicity of compounds was predicted using the ProTox-II web server [19]. Some aspects of compounds’ toxicity were predicted as follows: (i) the potential to induce endocrine disruptions was addressed using the Endocrine Disruptome web server [20], and (ii) cardiac toxicity was addressed using the Pred-hERG4.2 web server [21]. All these predictions were performed based on the SMILES structures of compounds.

#### 2.2.3. Prediction of Compounds’ Biological Activities

**Figure 1:**
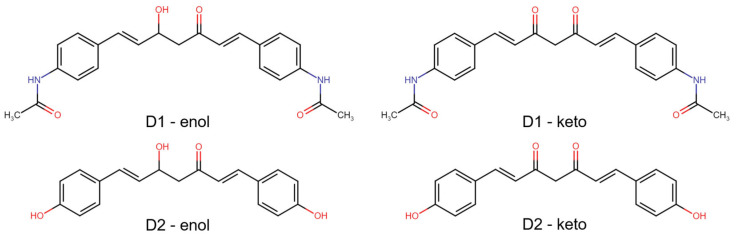
2D structures of the analyzed compounds—D1 and D2, in both enol and keto forms.

In predicting the biological activity of compounds, we initially calculated their bioactivity scores as G protein-coupled receptor ligands, ion channel modulators, nuclear receptor ligands, kinase, protease, and enzyme inhibitors using Molinspiration Cheminformatics tools [22]. The prediction of activity spectra for substances (PASS) was predicted using the PASS Online web server [23]. The molecular targets of analyzed compounds in both enol and keto forms (Figure 1), as well as the association of targets with diseases, were predicted using the Super-PRED web server [24]. 

### 2.3. In Vitro Biological Tests

#### 2.3.1. Cell Culture and Exposure to Curcumin Derivatives

Normal human lung fibroblasts (MRC-5 cell line) purchased from the American Type Culture Collection (ATCC, catalog number CCL-171) and cervix cancer cells (HeLa cell line from ATCC, catalog number CCL-2) were used to test the in vitro cytotoxicity of curcumin derivatives. The cells were grown at 37 °C in complete Dulbecco’s Modified Eagle’s Medium supplemented with 10% fetal bovine serum in a humidified atmosphere with 5% CO_2_. For cell detachment, a solution containing 0.25% trypsin and 0.53 mM EDTA was used. The cells were seeded in 96-well plates (for biocompatibility assessment) or 25 cm^2^ flasks (for oxidative stress analysis) at a cell density of 2 × 10^4^ cells/cm^2^ and left to adhere overnight. Then, the cells were exposed to increasing doses (1, 10, 50, and 100 µg/mL) of the two symmetric curcumin derivatives for 24 and 72 h. The tested solutions were previously sterilized under UV light for 3 h. The cells grown in curcumin-free culture medium were used as controls for each test. At the end of each incubation time, the cells were examined on an inverted fluorescence microscope Olympus IX71 (Olympus, Tokyo, Japan). 

#### 2.3.2. Cell Viability Assay

The 3-(4,5-dimethylthiazol-2-yl)-2,5-diphenyltetrazolium bromide (MTT, Sigma-Aldrich, St. Louis, MO, USA) assay, which is based on the mitochondrial succinate dehydrogenase activity in the viable cells, was used to quantify the cellular proliferation. The growth medium was removed after 24 and 72 h of incubation, and the cells were then incubated with 1 mg/mL MTT solution for 4 h at 37 °C. The purple formazan crystals generated in the viable cells were dissolved with 2-propanol (Sigma-Aldrich, USA), and the absorbance at 595 nm was measured using a FlexStation 3 multi-mode microplate reader from Molecular Devices (San Jose, CA, USA). Compound concentrations that produced 50% cell growth inhibition (IC50) were calculated from curves constructed by plotting cell viability (%) versus concentration (µg/mL) using the Quest Graph™ IC50 calculator (AAT Bioquest, Pleasanton, CA, USA).

#### 2.3.3. Griess Assay

With the use of Griess reagent, which is a stoichiometric solution (*v*/*v*) of 0.1% naphthyl ethylenediamine dihydrochloride and 1% sulphanilamide in 5% H_3_PO_4_, the nitric oxide (NO) concentration in collected culture media was determined after 24 and 72 h of incubation. Since this molecule is produced and released during inflammation and apoptosis, increased NO levels are a result of cytotoxic effects. Molecular Devices’ FlexStation 3 multi-mode microplate reader (San Jose, USA) was used to measure the absorbance of the equimolar mixture made up of Griess reagent and cell culture medium at 550 nm. The results were expressed as relative to the control.

#### 2.3.4. Fluorescence Staining Assay

After 24 and 72 h of incubation with the curcumin derivatives, the culture medium was removed, and cells were washed with phosphate-buffered saline (PBS) and incubated with different fluorescent dyes for 30 min at 37 °C in a humidified atmosphere with 5% CO_2_. The calcein-AM and ethidium homodimer-1 from LIVE/DEAD™ Viability/Cytotoxicity Kit (Thermo Fischer, Waltham, MA, USA) were used to label the living and dead cells, respectively, according to the manufacturer’s indications. The images were captured on an inverted fluorescence microscope, an Olympus IX71.

### 2.4. Biochemical Tests

#### 2.4.1. Cell Lysate

After the incubation with curcumin derivatives, cell lysis was performed. The cells were harvested from the flasks using 0.25% trypsin—0.53 mM EDTA solution, centrifuged at 1500× *g* for 5 min at 4 °C. Then, they were resuspended in 0.2 mL of PBS and sonicated (three times for 30 s in each tube, on ice) using the Hielscher Ultrasonic UP50H ultrasonicator. Next, the cell lysates were centrifuged at 10,000× *g* for 10 min at 4 °C, and the supernatant was collected in Eppendorf tubes, on ice, and stored at −80 °C for further testing. The total protein concentration was determined by measuring the absorbance at 595 nm after using the Bradford reagent and a calibration curve with bovine serum albumin.

#### 2.4.2. Measurement of Catalase (CAT) Activity

The spectrophotometric method described by Aebi in 1974 was used to identify the level of catalase activity [25]. The change in absorbance at 240 nm was monitored for 1 min due to a decrease in the H_2_O_2_ quantity in the reaction medium containing 50 µL of total diluted protein extract and 600 µL of potassium phosphate buffer [25]. The absorbance variation at 240 nm (on the ordinate) was plotted against time (on the abscissa), and the specific activity expressed in katal/mg protein was calculated using the JASCO V-530 spectrophotometer (JASCO Co., Ltd., Kyoto, Japan).

#### 2.4.3. Reduced Glutathione (GSH) Level Determination 

The GSH level was determined with the Glutathione Assay Kit from Sigma (Sigma-Aldrich, St. Louis, MO, USA), according to the protocol provided by the manufacturer. The method involves the reduction of 5,5′-dithiobis-2 nitrobenzoic acid (DTNB) by GSH with the formation of 5-thio-2 nitrobenzoic acid (TNB). Cell lysates were diluted accordingly and deproteinized with 5% 5-sulfosalicylic acid (SSA) (1:1). This was followed by centrifugation at 3000× *g* for 5 min at 4 °C to remove precipitated proteins. A volume of 10 µL was taken from the supernatant, over which 150 µL of a mix made from 8 mL of buffer solution (100 mM potassium phosphate buffer, pH 7; 1 mM EDTA) and 228 µL of DTNB 1.5 mg/mL were added. The absorbance of the yellow product obtained from this reaction was measured at 412 nm on the Tecan GENios spectrophotometer (TECAN GENios, Grödig, Austria). In order to calculate the number of nmol GSH in the analyzed samples, an extrapolation on the curve with the GSH standard was performed, and the results were expressed in nmol/mg protein.

#### 2.4.4. Determination of Malondialdehyde (MDA) Level 

The MDA level was determined using the fluorometric method with thiobarbituric acid (TBA). For this determination, a standard solution of malondialdehyde (1,1,3,3-tetramethoxypropane 1 mM) was used. Subsequently, 700 µL of 0.1 M HCl were pipetted over the MDA calibration curve and the cell lysates. The mixtures were homogenized and incubated at room temperature for 20 min. Further, a volume of 900 μL of 0.025 M TBA was added to all tubes and incubated for 65 min at 37 °C. During that time, the TBA-MDA products were formed. Subsequently, the fluorescence recorded using a Jasco FP-6300 fluorometer (JASCO Co., Ltd., Kyoto, Japan) was converted to nmoles malondialdehyde (MDA) using a 1,1,3,3-tetramethoxypropane standard curve.

#### 2.4.5. Western Blot

The protein level of heat shock proteins (HSP60 and HSP70) and minichromosome maintenance complex 2 (MCM2) was measured after 24 and 72 h of HeLa cells incubation with 50 µg/mL derivatives. Samples corresponding to 100 µg of protein were separated on a 10% SDS/PAGE under reducing conditions and transferred to 0.4 µm (Millipore) polyvinylidene difluoride (PVDF) membrane in a wet transfer system (Bio-Rad, Hercules, CA, USA). Membranes were blocked with the blocking solution included in the Western Breeze Chromogenic kit (Invitrogen) for 30 min at room temperature. Detection was performed with mouse monoclonal anti-HSP60, anti-HSP70, and anti-MCM2 primary antibodies (1:250 dilution Santa Cruz Biotechnology). Further, the membranes were processed according to the manufacturer’s instructions, using alkaline phosphatase-conjugated anti-mouse and anti-rabbit secondary antibodies and 5-bromo-4-chloro-3′-indole phosphate/nitroblue tetrazolium as a chromogenic substrate (Invitrogen by Thermo Fisher Scientific). The resulting bands were visualized and photographed using a transilluminator (ChemiDoc MP Video Documentation System, Bio-Rad) and were densitometered using the GelQuant.NET software version 1.7.8.

### 2.5. Statistical Analysis

All tests were statistically analyzed using a Student’s *t*-test (Microsoft Excel) and expressed as mean value ± standard deviation (SD) (*n* = 3 independent experiments, each of these including three technical replicates). A value of *p* less than 0.05 was considered statistically significant.

## 3. Results

### 3.1. Characterization of Symmetric Curcumin Derivatives by Spectrophotometric Methods

Two curcumin derivatives (D1—(1E,6E)-1,7-bis(4-acetamidophenyl) hepta-1,6-diene-3,5-dione and D2—(1E,6E)-1,7-bis(4-hydroxyphenyl) hepta-1,6-diene-3,5-dione (also known as bisdemethoxycurcumin or p, p-dihydroxy di cinnamoyl methane), that were produced using a less expensive and more environmentally friendly method than the traditional solvent-based one were utilized in this study. The synthesis and characterization of these curcumin analogs were described in more detail in our previous work [11]. Table 1 summarizes the characteristics of the compounds obtained by microwaves in higher yields and with shorter reaction times. 

**Figure 2:**
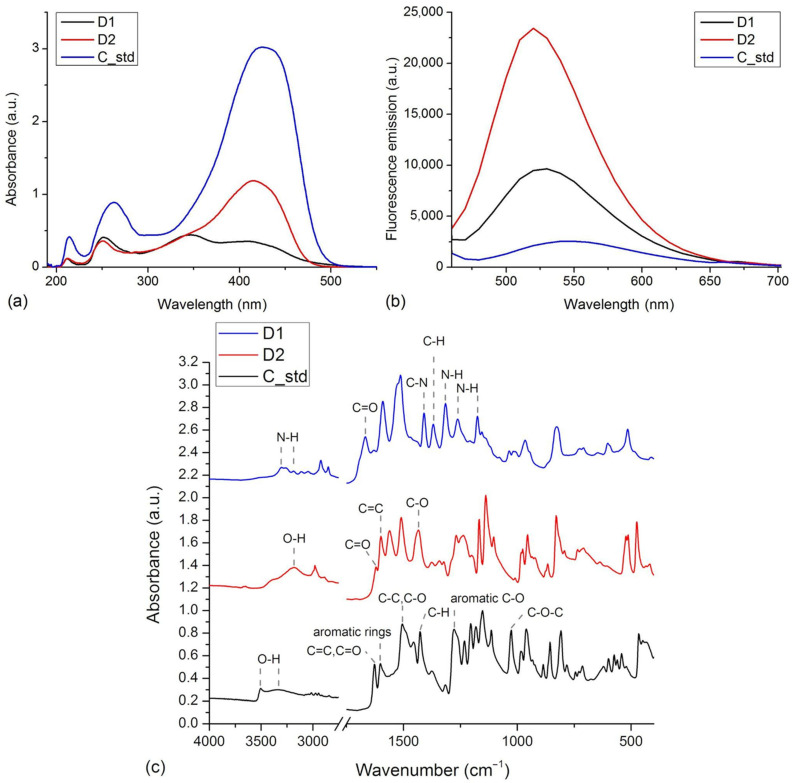
(**a**) The normalized UV-VIS spectra of D1, D2, and curcumin standard (C_std) in methanol. (**b**) Fluorescence spectra of D1, D2, and C_std samples, λex = 435 nm. (**c**) FTIR spectra of D1, D2, and curcumin standard (C_std) measured in the 4000–400 cm^−1^ range.

The UV-VIS measurements showed that samples dissolved in methanol, a polar solvent, absorb both in UV and VIS regions (Figure 2a). The samples present different absorbance intensities, with the curcumin standard presenting the highest absorption. The differences in absorption peaks are due to the electronic structure of compounds that is influenced by the functional groups attached to the aromatic rings [11]. In the UV region, the curcumin standard presents maxima at 214 nm and 263 nm. These are blue-shifted in the spectra of derivatives to 213 nm and 251 nm in D1 and 212 nm and 251 nm in the case of D2. Only in the case of D1 do we see an additional maximum at 347 nm. The curcumin standard presents a VIS absorption peak at 425 nm. The VIS absorption maxima of D1 and D2 are blue-shifted relative to the maximum of the standard, to 415 nm for D2 and 408 nm for D1. 

The fluorescence emission of the curcumin standard and the two derivatives were measured considering an excitation wavelength of 435 nm (Figure 2b). D2 presents the highest fluorescence intensity, followed by D1 and the curcumin standard. The maximum fluorescence emission of samples occurred at 550 nm for the curcumin standard, 530 nm for D1, and 520 nm for D2. The blue shifts in maximum fluorescence emission wavelengths seen in the case of derivatives are in agreement with the blue shifts in absorbance wavelengths. 

The FTIR spectra recorded on the compounds are presented in Figure 2c. The spectra show notable differences in agreement with the particular chemical structure of the compounds. These present many absorption bands due to the functional groups present in the structures and to the tautomerism of curcumin and derivatives, both enol and keto forms being present in the sample [26,27]. The spectrum measured on curcumin standard presents absorption peaks associated with its specific functional groups: 1601 cm^−1^ (aromatics rings vibrations), 3504, 3340 cm^−1^ (O-H stretching), 1278 cm^−1^ (aromatic C-O stretching), 1627 cm^−1^ (C=O and C=C vibrations), 1505 cm^−1^ (C-O and C-C vibrations), 1427 cm^−1^ (C-H bending), 1027 cm^−1^ (C-O-C stretching) [28]. The FTIR spectrum of D1 lacks the features related to the phenol or methoxy groups seen in the case of curcumin. Instead, it presents the contribution of acetamido-functional groups as N-H stretching at 3304 and 3185 cm^−1^, C-N stretching at 1315, 1262, and 1175 cm^−1^, C=O stretching at 1667 cm^−1^ and C-H bending at 1369 cm^−1^ [29]. Relative to the curcumin standard, the D2 derivative lacks the methoxy functional groups, which is reflected by the spectrum that lacks those contributions. The spectrum of D2 presents the contributions of phenol groups as O-H stretching at 3181 cm^−1^. In addition, the spectrum includes C=O stretching vibrations at 1624 cm^−1^, aromatic C=C stretching vibrations at 1599 cm^−1^, and in-plane bending of enol C-O at 1434 cm^−1^ [30].

### 3.2. Predictions of ADME/Drug-Likeness Parameters

#### 3.2.1. Drug-like Profile and Bioavailability 

The structures of analyzed compounds are presented in Figure 1. Their physicochemical properties are shown in Table 2. These properties, along with other properties like the number of atoms, number of rings, number of carbon atoms, and number of heteroatoms (which can be derived from the structures in Figure 1), were compared to the appropriate ranges for drug-likeness, resulting that both compounds, in enol and keto forms, presented no violations of the five drug-likeness rules (Table S1).

**Figure 3:**
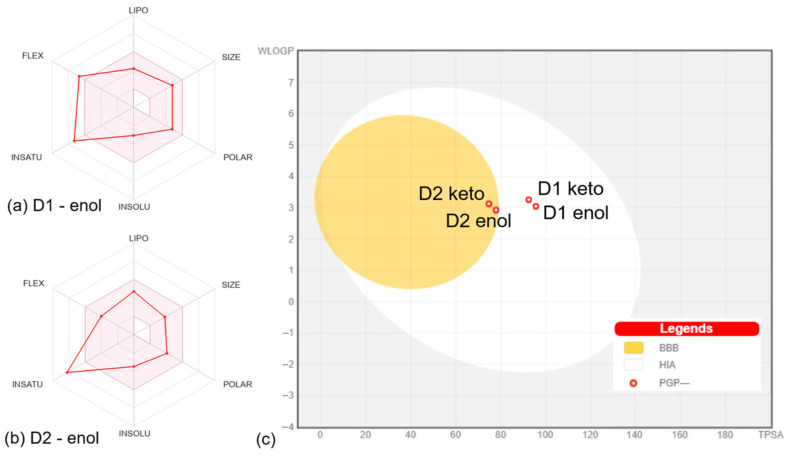
Bioavailability radar charts for D1 (**a**) and D2 (**b**) compounds in enol form. The space of physicochemical properties optimal for oral bioavailability is highlighted in pink. The values specific to the compounds are marked with red. (**c**) Boiled egg plot for all compounds. The white corresponds to the physicochemical space of highly probable passive gastrointestinal absorption (HIS), and the yolk corresponds to the space of probable blood-brain barrier (BBB) permeation. The red circles mark that compounds are not substrates for P-glycoprotein (PGP-). All plots were generated by SwissADME [12].

The properties defining the compounds’ lipophilicity (Log P as XLOGP3), size (molecular weight), polarity (total polar surface area), insolubility (Log S), unsaturation (Fraction Csp3), and flexibility (number of rotatable bonds) were compared to the suitable properties for oral bioavailability [12]. The D1 derivative in enol and keto forms presents two violations of the rules of flexibility (number of rotatable bonds > 9) and unsaturation (fraction Csp3 < 0.25), while the D2 derivative in enol and keto forms has one violation of unsaturation rules (fraction Csp3 < 0.26). The bioavailability radar charts for D1 and D2 compounds in enol form are presented in Figure 3a,b. The plots of compounds in keto form are similar to the results for enol form, showing the same violations of the rules. Moreover, both compounds in enol and keto forms present a bioavailability score of 0.55, which is found in the optimum range [31], suggesting that all compounds should present good bioavailability. 

The predictions performed with the SwissADME web server [12] included the pan-assay interference compounds (PAINS) implemented based on the approach of Baell et al. [32]. The analysis revealed zero alerts, suggesting that D1 and D2 are not promiscuous compounds expected to elicit strong responses regardless of the target. In what concerns the structural alerts implemented based on the work of Brenk et al. [33], an analysis that identifies fragments associated with poor pharmacokinetics, it was shown that D1 and D2 in enol form present one alert (michael_acceptor_1), while D1 and D2 in the keto form present two alerts (michael_acceptor_1 and beta_keto_anhydride). According to the lead-likeness rules (molecular weight > 350 and rotors > 7) [34], the D2 compound in both enol and keto could be a candidate for chemical modifications that can optimize its physicochemical features. 

**Table 2:** The physicochemical properties of compounds were predicted using the SwissADME server [12].

Compound	MW	F. Csp3	NRB	HBA	HBD	MR	TPSA(Å^2^)	XLOGP3	WLOGP	MLOGP	Log S
D1 enol	392.45	0.17	10	4	3	115.36	95.5	1.81	3.05	2.11	−3.06
D1 keto	390.43	0.13	10	4	2	114.40	92.34	2.32	3.26	2.04	−3.37
D2 enol	310.34	0.11	6	4	3	90.78	77.76	2.74	2.93	2.20	−3.48
D2 keto	308.33	0.05	6	4	2	89.92	74.60	3.26	3.13	2.13	−3.8

Notes: MW is molecular weight (measured in g/mol); F. Csp3, Fraction Csp3; NRB, number of rotatable bonds; HBA, number of H-bond acceptors; HBD, number of H-bonds donors; MR, molecular refractivity; TPSA, total polar surface area; XLOGP3, log P_o/w_ calculated by XLOGP program [35], version 3.2.2; WLOGP, log P_o/w_ calculated by the approach developed by Wildman and Crippen [36] and MLOGP, log P_o/w_ calculated based on the method implemented by Moriguchi et al. [13,37]; Log S, water solubility.

#### 3.2.2. Pharmacokinetic Profile of Compounds

The ADME properties of compounds predicted by pkCSM [18] are presented in Table S2. Regarding the absorption of compounds, D2 in the enol and keto form presents a high CaCo2 permeability. This parameter predicts the intestinal permeability and efflux liability, as revealed by Caco2 screening assays [38]. Both compounds in enol and keto forms present high percentages of intestinal absorption. These results show that both compounds are readily absorbed at the intestinal level. 

The distribution of compounds was addressed by calculating the parameters—steady-state volume of distribution (VDss), BBB permeability, and CNS permeability. VDss is a reliable parameter that describes the molecular tissue binding of drugs at equilibrium [39]. Higher VDss values suggest that a drug is distributed in tissues rather than plasma [18,40]. The values obtained for the analyzed compounds show that D2 and D3 in enol form have medium VDss values, while the keto forms of compounds present low VDss values. According to the values in Table S2, D1 and D2 compounds in enol and keto forms would present a mean distribution to the central nervous system (CNS). The Boiled-Egg plot [41] generated by SwissADME [12] based on WLOGP and TPSA properties (Figure 3c) predicts that D1 in enol and keto forms present suitable intestinal absorption and no BBB permeability, while D2 in enol and keto forms presents suitable intestinal absorption and BBB permeability. The BBB permeability is an important parameter since compounds able to permeate the CNS can be used as therapeutic agents in CNS diseases, but at the same time, such compounds could mediate secondary effects at the CNS level [42]. The plot in Figure 3c also shows that the compounds should not be P-glycoprotein substrates. P-glycoprotein appears to be involved in the efflux of several drugs like those prescribed in cancer therapy, bacterial infections, or psychiatric conditions [43,44,45].

Regarding the metabolism of the compounds, these mostly appear to be substrates for CYP3A4 (except for D2 in enol form) (Table S2). At the same time, the compounds were predicted as inhibitors for other cytochrome P450 (CYP) isoforms like CYP2C19, CYP2C9, and CYP3A4. CYP inhibition underlines pharmacokinetic drug-drug interactions that can result in serious side effects [46]. Therefore, the compounds analyzed here could be involved in drug-drug interactions, suggesting a difficult administration to patients with comorbidities. 

The compounds were not predicted to be substrates of organic cation transporter 2 (OCT2), suggesting that compounds have no potential for adverse reactions when they are administered with OCT2 inhibitors [18,40]. The total clearance values, considering both renal and hepatic clearance, are given in Table S2. The D1 compound in the enol and keto forms present larger values than the D2 compound in corresponding forms.

#### 3.2.3. Predicted Toxicity of Compounds

The predicted toxic doses (LD50), as determined using the ProTox-II web server [19], were 3200 mg/kg in the case of D1 enol or keto, 2000 mg/kg in the case of D2 enol and 2650 mg/kg for D2 keto. Based on these values, D1 in both forms and D2 in keto form is placed in the toxicity class V (may be harmful if swallowed). D2 in enol form was placed in toxicity class IV (harmful if swallowed). 

The toxicity features of compounds are presented in Table S3. Four classes of toxicity targets were addressed, namely organ toxicity, toxicity endpoints, nuclear receptor signaling pathways, and stress response pathways. The predictions suggest that D1 in enol and keto forms are inactive on all considered targets. The D2 compound appears inactive on most targets. D1 appears active on the mitochondrial membrane potential, a target involved in the stress response pathways. The toxicity of drugs at the mitochondrial level can manifest through the alteration of membrane potential that leads to inefficient harvesting of energy and reduction of cell viability [47]. This is a side effect to be considered, especially in the case of patients with decreased mitochondrial function [47]. At the same time, therapeutic strategies that selectively disrupt the mitochondrial membranes of senescent or diseased cells are being developed [48]. Also, D2 appears active on estrogen receptor alpha, a target belonging to nuclear receptor signaling pathways. Estrogen receptor alpha is usually a primary target for toxic compounds causing endocrine system disruptions [49]. Given this potential of the D2 compound, we performed additional predictions on the disruptive effect of compounds on the endocrine system. 

The probabilities of compounds binding at 18 human nuclear receptors crystal structures were predicted using a molecular docking-based platform called Endocrine Disruptome [20]. The results are presented in Figure S1. The compounds do not possess a high probability of binding at any of these receptors. D1 in enol form presents a low probability of binding at 14 target structures and a medium probability (yellow) of binding at four receptors. D1 in keto form presents a medium binding probability for three targets, one of them being encoded with orange. This target is peroxisome proliferator-activated receptor alpha. In the case of the D2 compound, we notice a larger number of targets in the medium probability region. D2 in enol form presents medium binding probabilities for seven targets, two of which are encoded with orange, namely the androgen receptor and mineralocorticoid receptor. D2 in keto form also presents medium affinity for 7 targets, from which 3 are encoded with orange—the androgen receptor, the mineralocorticoid receptor, and the thyroid hormone receptor alpha. In the current prediction, D2 in both forms appears to present a medium (yellow) affinity for estrogen receptor beta. Overall, these predictions indicate that the D2 compound have a higher affinity for nuclear receptors relative to D1.

In what concerns the cardiotoxicity of the compounds, the Pred-hERG4.2 web server [21] was used to predict their ability to modulate hERG ion channels and to determine their structural features that most contribute to the blockade. hERG are cardiac ion channels whose inhibition is linked to arrhythmia, being major targets in addressing the drugs’ toxicity [50]. The predictions have shown that D1 in enol form has cardiotoxic potential (50% confidence level), D1 in keto form has non-cardiotoxic potential (60% confidence level), D2 in enol form has non-cardiotoxic potential (50% confidence level), and D2 in keto form has non-cardiotoxic potential (70% confidence level). Figure S2 shows the structural features that contribute to these properties. D1, in enol form, the only potential cardiotoxic compound, presents regions with high positive contributions to the blockade and no regions with negative contributions to the hERG blockade. 

#### 3.2.4. Predicted Biological Activity of Compounds

The biological activities of compounds were initially predicted using Molinspiration Cheminformatics tools [22] (scores presented in Table S4). The platform predicts bioactivity scores considering the major targets of G-protein coupled receptors (GPCR), ion channels, kinases, nuclear receptor ligands, proteases, and enzymes. Active molecules present scores greater than 0.0, moderately active compounds present scores in the −0.5 to 0.0 range, and inactive molecules present scores below −0.5 [51]. D1 in enol forms appears active on GPCRs, proteases, and enzymes and moderately active on the other targets. D1 in keto form is moderately active on all targets. D2 compound in enol form could be active as a GPCR ligand, nuclear receptor ligand, protease, and enzyme inhibitor, and medium active on ion channels or kinases. D2 in keto form is active on nuclear receptors and enzymes and moderately active on ion channels, kinases, and proteases. 

The Activity Spectra for Substances (PASS) prediction has led to the identification of biological activities of compounds regarding the pharmacological activity, the mechanism of action, toxic outcomes, the impact on metabolic enzymes, transporters, and gene expression [23]. For each prediction, the server estimates predicted activity (Pa) and predicted inactivity (Pi) scores. The server predicted 1067 activities for D1 in enol form, from which 55 activities presented Pa > 0.5 and 3 activities presented Pa > 0.8. In the case of D1 in keto form, 1245 activities were predicted, from which 54 presented Pa > 0.5, and 4 presented Pa > 0.8. D2 in enol form led to the identification of 1860 activities—160 with Pa > 0.5 and 26 with Pa > 0.8. D2 in keto form was associated with 2109 activities—284 with Pa > 0.5 and 31 with Pa > 0.8. These predictions show that D2 presents more predicted biological activities, including more of the high score predicted activities. D2 in keto form appears to be the most active. From the list of activities, we selected some relevant to cancer development and treatment: (i) D1 in enol form: apoptosis agonist (Pa = 0.676, Pi = 0.017), anti-inflammatory (Pa = 0.595, Pi = 0.033); (ii) D1 in keto form: apoptosis agonist (Pa = 0.673, Pi = 0.018), anti-inflammatory (Pa = 0.607, Pi = 0.030), antineoplastic—multiple myeloma (Pa = 0.560, Pi = 0.006), radiosensitizer (Pa = 0.561, Pi = 0.025); (iii) D2 in enol form: apoptosis agonist (Pa = 0.854, Pi = 0.005), anti-inflammatory (Pa = 0.718, Pi = 0.014), anticarcinogenic (Pa = 0.647, Pi = 0.011), radioprotector (Pa = 0.621, Pi = 0.014), radiosensitizer (Pa = 0.609, Pi = 0.013), antimutagenic (Pa = 0.600, Pi = 0.010), antioxidant (Pa = 0.576, Pi = 0.005); (iv) D2 in keto form: preneoplastic conditions treatment (Pa = 0.912, Pi = 0.002), apoptosis agonist (Pa = 0.871, Pi = 0.005), antimutagenic (Pa = 0.790, Pi = 0.004), anti-inflammatory (Pa = 0.704, Pi = 0.015), radiosensitizer (Pa = 0.666, Pi = 0.006), antioxidant (Pa = 0.637, Pi = 0.004), antineoplastic (Pa = 0.619, Pi = 0.041), radioprotector (Pa = 0.566, Pi = 0.019), anticarcinogenic (Pa = 0.555, Pi = 0.015). These biological activities were predicted with high Pa values and very low Pi values (close to zero), suggesting that the compounds could exert these activities. 

The molecular targets of compounds were predicted using a Super-PRED web server [24]. From the high probability targets (probability > 80%), we selected those targets with indications of human diseases. It can be seen in Table 3 that some targets are predicted for all compounds, like DNA-(apurinic or apyrimidinic) lyase or DNA topoisomerase II alpha, while some targets are specific only for D3 in enol form, such as cathepsin D, transthyretin, estrogen receptor beta and C-X-C chemokine receptor type 4. The identified targets have indications in different types of cancers, as well as in other diseases like neurodegenerative, psychiatric, metabolic, or cardiovascular maladies. 

### 3.3. In Vitro Biological Evaluation

#### Evaluation of Cytotoxicity Induced by Curcumin Derivatives

**Figure 4:**
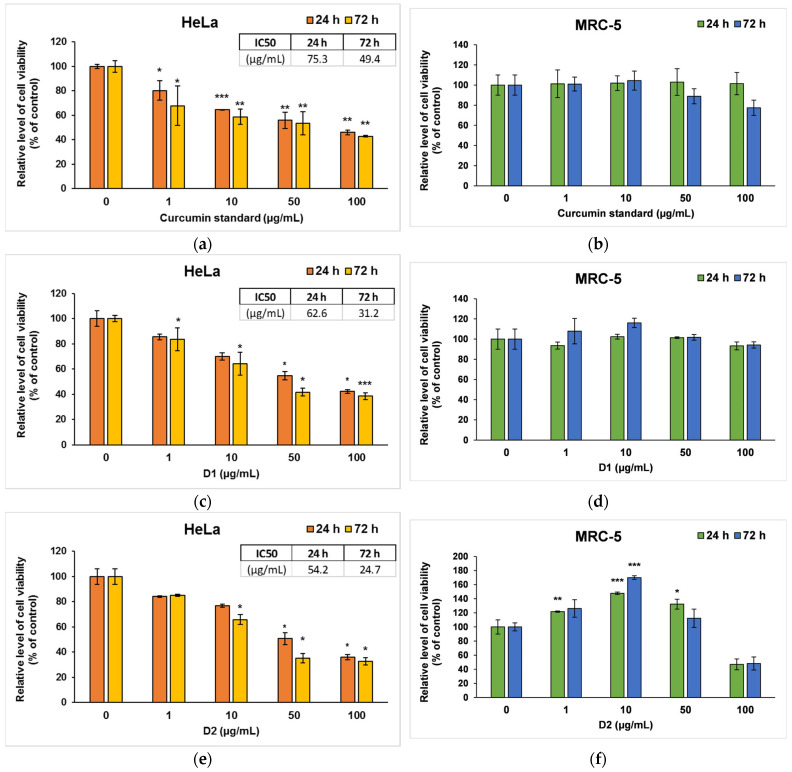
Cell viability measured by MTT assay after the 24- and 72-h incubation of HeLa cancer cells (**a**,**c**,**e**) and MRC-5 non-tumoral cells (**b**,**d**,**f**) with curcumin standard (**a**,**b**), D1 (**c**,**d**) and D2 (**e**,**f**) curcumin derivatives. Results are expressed as means ± standard deviation (SD) (*n* = 3) and represented relative to the untreated cells (control). * *p* < 0.05, ** *p* < 0.01 and *** *p* < 0.001 compared to control.

In order to highlight a specificity of action on malignant cells, HeLa tumor cervix cells and MRC-5 non-tumor cells were exposed to different concentrations of standard curcumin and two symmetric curcumin derivatives for up to 72 h. First, the cell viability was determined using the MTT assay, which provides information on the proliferation, survival, and metabolic activity of cells and the cytotoxic effects of these analogs on HeLa and MRC-5 cell lines, as shown in Figure 4.

In the case of curcumin standard, a dose-dependent decrease compared to control was observed for HeLa cancer cells (Figure 4a), but no significant changes were recorded on MRC-5 cells (Figure 4b). Similarly, D1 managed to decrease the viability of tumor cells by almost 50% of the control level at concentrations of 50 and 100 μg/mL (Figure 4c), while no major changes were noticed in normal MRC-5 cells (Figure 4d). In contrast, D2 induced a significant decrease in the number of both types of cells compared to the control at the highest concentrations used of 100 µg/mL (Figure 4e,f). But interestingly, lower doses (1 or 10 μg/mL) of D2 increased cell viability in non-tumor cells. This phenomenon could be cell-specific since fibroblasts are normally involved in wound healing and tissue remodeling processes. The hormetic behavior of curcumin (stimulatory at low doses and inhibitory at high doses) in human skin cells was also mentioned in other in vitro studies [52,53]. The potential to selectively stimulate apoptosis of cancer cells with minimum effects on normal cells was also previously described for curcumin and its other various derivatives [54,55]. The IC50 values (Figure 4a,c,e), obtained from cell survival plots on HeLa cells, were lower in the case of both derivatives compared to the curcumin standard, proving their higher antitumor efficiency.

**Figure 5:**
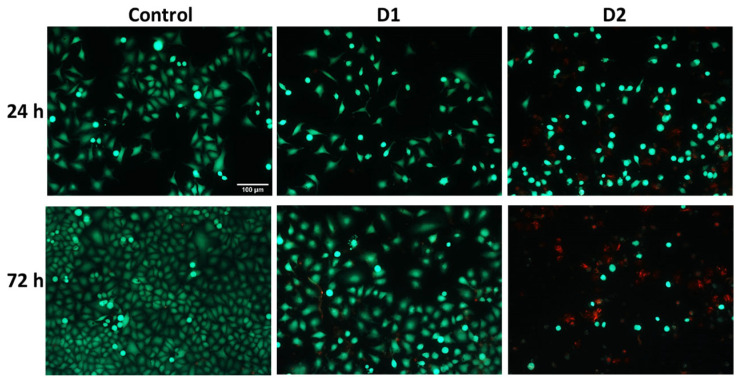
Representative fluorescence microscopy images were obtained using the Live & Dead assay after the 24- and 72-h exposure of HeLa human cervical cancer cells to 50 µg/mL concentration of curcumin derivatives (D1 and D2). Viable cells are stained green with calcein AM, and dead cells are stained red with ethidium homodimer. The control is represented by untreated HeLa cells. Images were obtained on an Olympus IX71 inverted microscope, 10× objective. The scale bar (100 µm) is valid for all images.

Visualization of live and dead cells was highlighted using the Live & Dead test (Figure 5), selecting only the concentration of 50 µg/mL for the two tested derivatives based on the results obtained from the MTT test. It can be observed that the viability of HeLa cells was affected in the case of 72-h exposure to the curcuminoid derivatives compared to the first incubation interval, and the greatest cytotoxic effect was noted in the case of the D2 derivative. These findings confirmed the results of the MTT assay presented in Figure 4.

**Figure 6:**
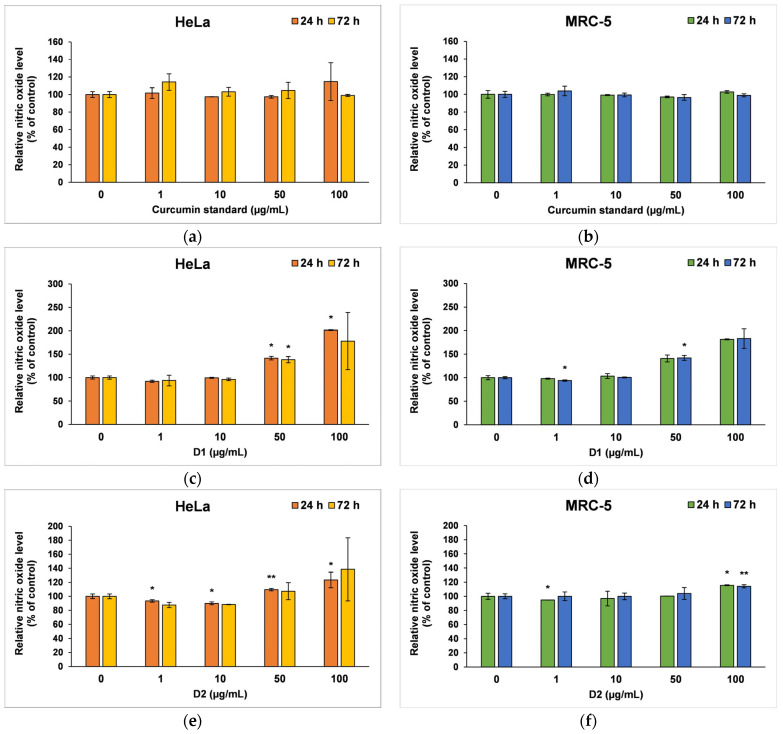
Nitric oxide level measured by Griess assay after the 24- and 72-h incubation of HeLa cancer cells (**a**,**c**,**e**) and MRC-5 non-tumoral cells (**b**,**d**,**f**) with curcumin standard (**a**,**b**), D1 (**c**,**d**) and D2 (**e**,**f**) curcumin derivatives. Results are expressed as means ± standard deviation (SD) (*n* = 3) and represented relative to the untreated cells (control). * *p* < 0.05 and ** *p* < 0.01 compared to control.

The amount of NO released in the culture medium (Figure 6) revealed the degree of cytotoxicity induced by the curcumin, being also a marker for inflammatory processes. The curcumin standard was well tolerated by both cell types; therefore, no major changes were recorded compared to the control after 24 or 72 h of incubation (Figure 6a,b). In contrast, at higher concentrations of the D1 derivative (50 and 100 µg/mL), a similar inflammatory potential was observed in both tumor cells and normal fibroblasts (Figure 6c,d). A ~40% increase in NO level above control was reported after 24 or 72 h of incubation in the presence of 50 µg/mL of this curcuminoid. In addition, NO levels doubled when 100 µg/mL of D1 was tested for 24 and 72 h. The D2 derivative did not increase the release of NO in the medium of HeLa or MRC-5 cells compared to control when it was incubated at concentrations lower than 100 µg/mL (Figure 6e,f). However, concentrations of 100 µg/mL raised the NO level by 38% above the control of HeLa cells and only 15% above the MRC-5 control cells after 72 h, this difference between the two cell lines being in agreement with the results of the MTT assay. 

### 3.4. Oxidative Stress and Antioxidant Defense

Based on the cell viability results, only the concentrations of 10 and 50 µg/mL were selected as representative for further biochemical investigations that included catalase (CAT) activity, reduced glutathione (GSH) concentration, and malonaldehyde (MDA) level in both cell lines. Also, because the toxicity mechanism of curcumin is very well known, the following tests only focused on the effects induced by the two synthesized derivatives (D1 and D2). 

**Figure 7:**
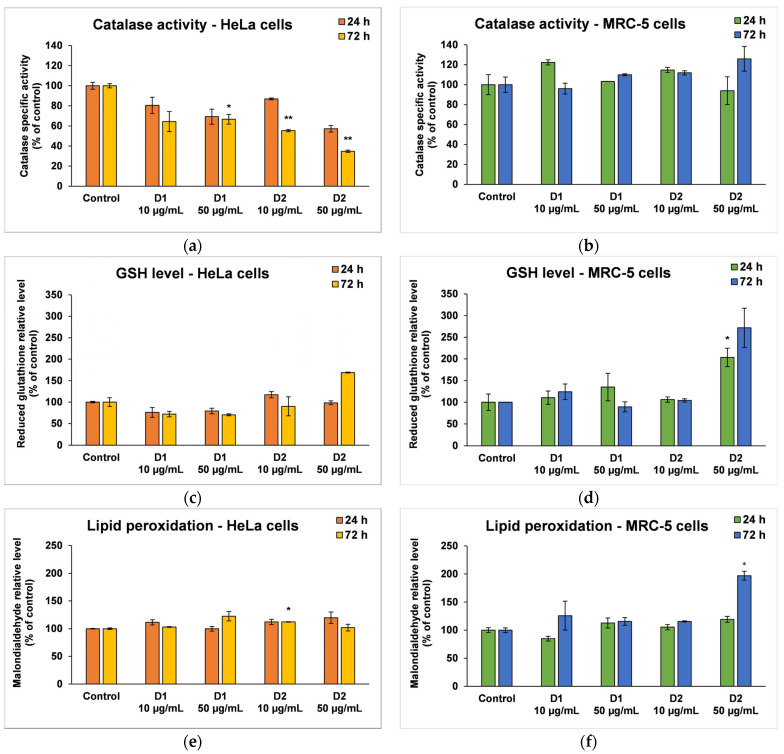
Oxidative stress and antioxidant defense as shown by relative levels of catalase-specific activity (**a**,**b**), reduced glutathione (**c**,**d**), and lipid peroxidation (**e**,**f**) in HeLa cancer cells and MRC-5 non-tumoral cells incubated for 24 and 72 h with 10 and 50 μg/mL of D1 and D2 curcumin derivatives. Results are expressed as means ± standard deviation (SD) (*n* = 3) and represented relative to the untreated cells (control). * *p* < 0.05 and ** *p* < 0.01 compared to control.

CAT is an intracellular antioxidant enzyme that breaks down oxygen peroxide (a dangerous compound that can react with biomolecules, affecting their structure and function) into oxygen and water. No major changes were noticed in the case of MRC-5 cells, but the results were significant for HeLa cells after 72 h (Figure 7a,b). A significant decrease in CAT activity (by 36% and 64%, respectively, compared to the control) was induced following the incubation of HeLa cells with a concentration of 50 µg/mL D1 and D2.

GSH is the major antioxidant compound in human cells that is able to react with xenobiotics directly or through enzyme-catalyzed reactions. In cancer cells, the level of GSH increases in order to protect the cells from damage caused by free radicals, peroxides, and toxins, as well as to maintain the redox state. In the MRC-5 cells treated with the D1 curcumin derivative, the GSH level was not significantly modified, but it decreased in HeLa cells compared to the control (Figure 7c,d). A 30% decrease in GSH levels from control was recorded in the case of 50 µg/mL D1 after 72 h of incubation. This suggests that the antioxidant defense was altered, and the cells were exposed to oxidative stress and initiated cell death. In contrast, the highest dose of D2 (50 µg/mL) increased GSH levels in both types of cells as a result of antioxidant defense against ROS-induced oxidative stress.

**Figure 8:**
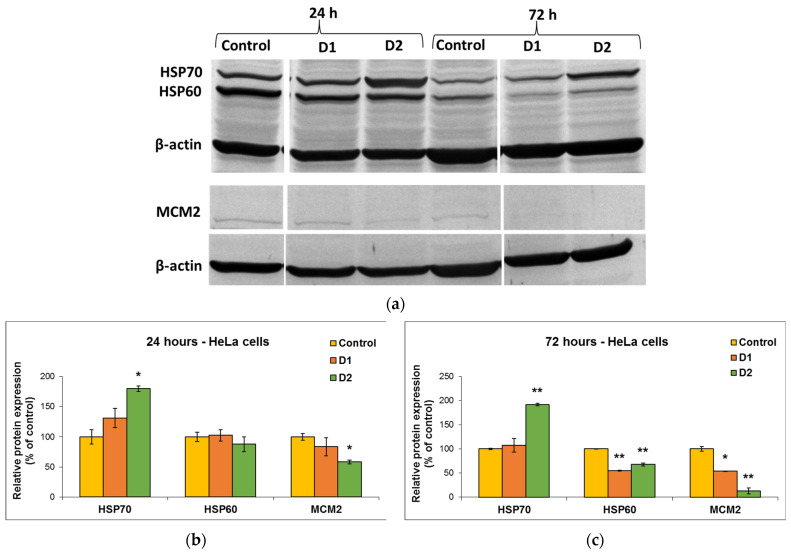
Representative Western Blot membrane (**a**) showing HSP70, HSP60, and MCM2 protein bands after exposure of HeLa human cervical cancer cells for 24 (**b**) and 72 (**c**) h to 50 µg/mL of D1 and D2 curcumin derivatives. The control is represented by untreated HeLa cells. β-actin protein was used to normalize to the same amount of total protein in each sample. Results are expressed as means ± standard deviation (SD) (*n* = 3) and represented relative to the untreated cells (control). * *p* < 0.05 and ** *p* < 0.01 compared to control.

MDA is one of the end products of polyunsaturated fatty acid peroxidation in cells. Free radicals are those that generate the process of lipid peroxidation in an organism, and an increase in them causes the overproduction of MDA, its levels being a marker for oxidative stress and antioxidant status in cancer patients [56]. In HeLa cells, the MDA level did not change significantly after the incubation with D1 and D2 derivatives, only a slight increase of approximately 20% compared to the control being recorded at the concentration of 50 µg/mL (Figure 7e). In the case of MRC-5 cells, the MDA level increased by almost 100% over control after the exposure to 50 µg/mL of D2 derivative for 72 h (Figure 7f). This result was in agreement with the previous cytotoxicity findings. The levels of HSPs and MCM2 in HeLa cells after exposure to different concentrations of curcumin derivatives are shown in Figure 8. An increased level of HSP70 protein expression and a decreased expression of HSP60 and MCM2 proteins were revealed in cells incubated with D2 compared to control, especially after 72-h exposure. HSP70 is a chaperone protein and has a critical role in the survival and growth of cancer cells. Thus, the increased level obtained in the case of the HSP70 protein could suggest that HeLa tumor cells managed to maintain their viability by synthesizing this protein. A normal level of HSP70 protein protects non-tumor cells from apoptosis, but its overexpression is found in cancer cells that show resistance to chemotherapy, as evidenced by the incubation with the D2 derivative.

In the case of HSP60 and MCM2 protein expression, after the exposure of HeLa cells to curcumin derivatives for 24 h and 72 h, the levels decreased in comparison with the control. This explains why the tested curcumin derivatives had a high antitumor capacity on HeLa cells and were able to reduce their viability over 50% of control, as the decrease in the HSP60 protein level is associated with the suppression of cancer cell proliferation. Also, the major decrease in the level of MCM2 protein indicated the end of the cell cycle and cell division.

## 4. Discussion

Despite the medical progress and new therapeutic strategies that have been successfully developed in the last decades of research, drug resistance continues to be the main limiting factor in curing cancer. Phytochemicals and their derivatives with strong antioxidant, anti-inflammatory, and antitumoral effects provide hope for increasing cancer patients’ treatment efficacy and reducing side effects [57]. Since 1985, when Kuttan et al. [58] mentioned the antitumor potential of curcumin for the first time, its pharmacological effect on various forms of cancer has been confirmed by numerous studies [59,60]. Moreover, its role in advanced cancer treatment and supportive care is currently addressed in phase III clinical trials after the promising results obtained in numerous phase I–II trials [61]. But, as mentioned before, the main problem of curcumin is its low absorption and poor bioavailability [62]. There have been numerous attempts to modify the structure of curcumin, and thus, new derivatives have been synthesized to overcome these limitations and improve the anticancer activity of curcumin.

Within our work, the therapeutic potential of two symmetric β-diketonic compounds was evaluated by various in silico and in vitro studies. The two curcumin derivatives were obtained by microwave irradiation as an energy- and time-saving process and a sustainable method related to green chemistry. Relative to curcumin, the D1 compound presents an amide group symmetrically grafted onto the structure of the aromatic residues at the end of the β-diketonic aliphatic chain. Theoretical and practical studies in recent years have confirmed the potential of compounds containing nitrogen atoms in their structure as having an antiproliferative potential in a variety of cancer cell lines, and the amide group is one of the forms in which the nitrogen atom can be generally bound in the organic molecule [63,64]. D2 compound has the structure of curcumin without the methoxy functional groups. The resulting compound, bis(demethoxy)curcumin, was reported to present higher stability and increased cellular uptake, along with antiproliferative and antimetastatic properties [30].

Curcumin and the synthesized compounds present keto-enol tautomerism. The two forms coexist in different ratios depending on the solvent in which they are dissolved. The estimation of the ratio between the tautomeric forms at equilibrium was achieved by UV-Vis spectral deconvolution using the FWHM (Full Width at Half Maximum) method [65]. The auxiliary groups symmetrically grafted onto the aromatic residues of the beta-diketonic system influence the keto-enol equilibrium of the two molecular structures. In the case of D1, the amide group is conjugated with the aromatic residue by giving up the electrons belonging to the nitrogen and favors the stability of the ketone form at 58% in the keto-enolic tautomer system [66]. While the hydroxyl groups grafted on the benzene residues of D2 and the polarity of the solvent favor the stability of the tautomer equilibrium towards the enolic form by establishing intramolecular hydrogen bonds, estimated at 82% compared to the ketone form in the system [66].

Given the structural differences of D1 and D2 compounds relative to curcumin and the fact that the D1 compound is not found in nature, we performed extensive bioinformatics predictions to understand the pharmacokinetic properties and toxicity profile of these compounds. Both keto and enol forms were considered in the predictions. In addition, theoretical studies were carried out to predict the targets of the compounds and the applicability of the identified targets in human diseases, especially cancer. Thus, our results (Table 3) predicted the antitumor activity of compounds D1 and D2, and the in vitro tests confirmed the antitumor activity of the curcumin analogs.

Furthermore, the compounds were predicted to be drug-like and to present good bioavailability, with no PAINS alerts. The targets predicted for both compounds were associated with neurodegenerative diseases. In addition, they do not appear to influence the nuclear receptor signaling pathways or the stress response pathways. An exception is the D2 derivative, which appears to modulate one target from each of these pathways. At the same time, the D2 compound appears to have a medium affinity for about half of the different targets considered in the endocrine disruption potential assessment. On the other hand, D1 in enol form is the only potential cardiotoxic compound.

The antitumor effect of curcuminoid derivatives has been demonstrated on several types of cancer cells, such as breast [67], prostate [68], lung [69], and colon [70], but there are very few studies on cervical cancer, the second most common cause of cancer death among women. One of the intriguing features of curcumin is its specific cytotoxicity against cancer cell lines, in contrast to normal cells. Chemotherapeutics and other phytochemicals rarely have this property. The selective effect of curcumin and its derivatives on tumor cells while sparing normal cells is not fully understood, but several possible reasons have been suggested. First, Kunwar et al. showed that cellular uptake of curcumin is higher in tumor cells than in normal cells [71]. Secondly, the malignant cells tend to have lower glutathione levels than normal cells, thus enhancing their sensitivity to curcumin [72]. Another possible explanation is that curcumin could interfere with multiple signaling pathways that are crucial for cancer cell survival, proliferation, and invasion. For this reason, we compared the cytotoxicity of the two derivatives using both types of cells: a tumor cell line (HeLa cells—human cervix) and a normal one (MRC-5—human lung fibroblasts).

The mechanism of action of curcumin analogs on cervical cancer is not yet fully known, but we tried to contribute to its elucidation by comparing it with that of curcumin, which has been extensively studied. The main effects of curcumin on cancers are the inhibition of cell proliferation, invasion, and migration, as well as the generation of cell apoptosis and autophagy.

Unrestricted cell proliferation resulting from dysregulation of the cell cycle is a significant factor in the development of tumors. Our results (Figure 4) showed that both derivatives inhibited the proliferation of cervix cancer cells in a dose-dependent manner but exhibited less toxicity against non-cancer cells. The most prominent effect on cell viability was observed for the D2 derivative (bisdemethoxycurcumin), which was more efficient than the curcumin standard, but it also induced a decrease by almost 50% of control in the viability of MRC-5 cells at the highest dose (100 µg/mL). The potent anti-proliferative effect of this derivative was confirmed by the Western blot results, showing a decreased expression of MCM2 protein compared to control cells. This protein is involved in cell cycle regulation and promotes the development of cervical cancer by inhibiting cell apoptosis. It was shown that the decrease in MCM2 protein level attenuated the proliferation of malignant cervical cells, as well as their transition from the G0/G1 phase to the S phase of the cell cycle. Silencing of the gene encoding the MCM2 protein-induced cell cycle arrest in the G0/G1 phase [73].

Another potential mechanism underlying the antitumor and chemosensitizing effects of curcumin on HeLa cells might be its modulatory effect on heat shock proteins (HSPs) [74]. Several studies have indicated the role of curcumin in the induction of HSP70 and inhibition of HSP60 expression in cancer [75,76]. It has been shown that HSP60 plays a central role in tumor cell maintenance by stabilizing the cell survival pathways and inhibiting the p53 protein function [77], while HSP70 is a chaperone protein and, therefore, has a critical role in the survival and growth of cancer cells. Thus, the increased level obtained in the case of the HSP70 protein indicated that HeLa tumor cells managed to maintain their viability by synthesizing this protein. A normal level of HSP70 protein protects non-tumor cells from apoptosis, but its overexpression is found in cancer cells that show resistance to chemotherapy, as evidenced after the incubation with the D2 derivative (Figure 8).

Its dual character allows curcumin to act both as an antioxidant and prooxidant, mediating a pro-apoptotic effect by inducing more ROS. It has been reported that curcumin can suppress cancer via cell apoptosis at concentrations over 20 μg/mL [78]. Singh et al. [79] reported that curcumin increased the inducible nitric oxide synthase (iNOS) expression in HeLa cells, leading to the inhibition of Ras and ERK pathway and, ultimately, causing the activation of apoptosis-inducing factor (AIF), release of cytochrome c and induction of apoptosis through the mitochondrial pathway. In order to assess the potential of D1 and D2 derivatives to induce oxidative stress in tumor cells but not in normal cells, CAT activity, GSH level, and MDA concentration were measured within this study. Our results (Figure 7a,b) showed that CAT activity decreased in HeLa cells after 72 h, while no major changes occurred in MRC-5 cells. A different effect was observed in the case of GSH level (Figure 7c,d). The D1 curcumin derivative induced a GSH depletion in cervical cells and no significant effects in lung fibroblasts, supporting the hypothesis that these derivatives induced oxidative stress only in the tumor cells. In contrast, the D2 derivative exerted an accumulation of GSH in both cell types. An increase in intracellular GSH levels has been linked with the suppression of CAT activity by most antitumoral agents [80]. Moreover, the level of MDA was not increased compared to the control in a significant way (Figure 7e), highlighting that the mechanism of action of these compounds does not target lipid peroxidation, at least for the concentrations and incubation times tested. These results are similar to those from previous studies, which showed that curcuminoids are potent in suppressing ROS-induced lipid peroxidation [81], but different from those showing that curcumin is a more potent antioxidant than desmethoxycurcumin or bisdemethoxycurcumin [82,83].

## 5. Conclusions

Obtaining new curcumin derivatives may help to improve the chemical and biological properties, targeting tumor cells with higher affinity. The two compounds, D1 and D2, in both their enol and keto forms, were extensively analyzed using bioinformatics methods. Their ADME profile showed that the compounds present a high intestinal absorption, with compound D2 having the ability to permeate through the BBB. Also, both compounds were predicted as inhibitors for some CYP enzymes, and none of them were predicted as hepatotoxic, carcinogenic, immunotoxic, mutagenic, or cytotoxic. Concerning the biological activity of these compounds, they were predicted to act as apoptosis agonists or anti-inflammatory agents. In the case of D2, many other potential activities were predicted, such as anticarcinogenic, antimutagenic, antineoplastic, radioprotector, or radiosensitizer activity. Overall, the bioinformatic analysis of compounds suggests that these could be used as drugs with relevant applications in cancer and even neurodegenerative diseases. The in vitro results obtained in the present work converge to the conclusion that the two studied curcumin derivatives had cytotoxic effects on HeLa tumor cells in relatively low concentrations, suggesting significant antitumor properties without irreversibly affecting normal cells. Taking into account their antioxidant and antitumor properties, these compounds have great prospects to be tested in vivo and subsequently used in the production of drugs with a targeted anticancer effect, as they present a specificity of action on malignant cells.

Our data also serves as a valuable foundation for the design of future improved products, allowing for the optimization of therapeutic properties, safety profiles, and delivery systems. Data on the structure-activity relationship (SAR) of curcumin derivatives helps in understanding how specific modifications affect their biological activity. This information is crucial for designing future derivatives with optimized chemical structures and enhanced therapeutic efficacy. On the other hand, our insights into the pharmacokinetics (ADME profile) of curcumin derivatives can guide efforts to improve bioavailability. Future products can be designed with modifications that enhance the compound’s absorption and systemic availability. This iterative process of research and development helps advance the field and offers innovative solutions for various health challenges.

## Figures and Tables

**Table 1:** Physicochemical characteristics of the curcumin derivatives obtained in the microwave field [11].

Compound	Molecular Formula	Color	Elemental Analysis (%)		
			Carbon	Hydrogen	Nitrogen
D1	C_23_H_22_N_2_O_4_	Dark orange	70.55	5.82	6.98
D2	C_19_H_16_O_4_	Yellow	73.85	5.36	-

**Table 3:** Molecular targets of compounds predicted with high probability (>80%) and model accuracy (>80%) using a Super-PRED web server [13]. The indications of identified targets in human disease were also predicted by the web server with high probability and model accuracy.

Target	Indications of Predicted Targets	Active Compounds
Pregnane X receptor	Arteriosclerosis	D1 enol form, D1 keto form, D2 enol form
DNA-(apurinic or apyrimidinic site) lyase	Glioma, Melanoma, Ocular cancer, Solid tumor/cancer	D1 enol form, D1 keto form, D2 enol form, D2 keto form
PI3-kinase p110-alpha/p85-alpha	Breast cancer, Follicular lymphoma, Non-Hodgkin lymphoma, Prostate cancer, Solid tumor/cancer	D1 enol form, D1 keto form,
DNA topoisomerase II alpha	Solid tumor/cancer	D1 enol form, D1 keto form, D2 enol form, D2 keto form
Serotonin 2c (5-HT2c) receptor	Alcohol dependence, Alzheimer’s disease, Anxiety disorder, Attention deficit hyperactivity disorder, Depression, Diabetic complications, Drug abuse, Dyskinesia, Generalized anxiety disorder, Hyperprolactinemia, Major depressive disorder, Metabolic disorder, Migraine, Mood disorder, Neurological disorder, Non-Hodgkin lymphoma, Obesity, Pain, Parkinson’s disease, Primary insomnia, Psychotic disorder, Schizophrenia, Sleep-wake disorder	D1 enol form, D2 enol form
Beta-glucuronidase	Mucopolysaccharidosis, Periodontal disease	D1 enol form, D2 enol form
Neuronal acetylcholine receptor; alpha4/beta4	Alzheimer’s disease, Aneurysm, Hypertensive emergency, Hypotension, Tobacco dependence	D1 enol form, D2 enol form
Cathepsin D	Hypertension, Multiple sclerosis	D2 enol form
Transthyretin	Amyloidosis, Cardiomyopathy, Hereditary amyloidosis	D2 enol form
Estrogen receptor beta	Alzheimer’s disease, Breast cancer, Carcinoma, Cushing disease, Estrogen deficiency, Hepatitis virus infection,	D2 enol form
C-X-C chemokine receptor type 4	Acute lymphoblastic leukemia, Acute myeloid leukemia, Autoimmune diabetes, B-cell chronic lymphocytic leukemia, Bone marrow transplantation, Breast cancer, Constitutional neutropenia, Hematological malignancy, Human immunodeficiency virus infection, Macular degeneration, Melanoma, Merkel cell carcinoma, Multiple myeloma, Myelodysplastic syndrome, Non-Hodgkin lymphoma, Pancreatic cancer, Peripheral vascular disease,	D2 enol form

## Data Availability

Data are available at request from the corresponding author.

## Supplementary Materials

The following supporting information can be downloaded at: https://www.mdpi.com/article/10.3390/pharmaceutics16020205/s1. Table S1. The acceptable values of compounds’ physicochemical properties according to the drug-likeness rules of Lipinski, Ghose, Veber, Egan and Muegge, and the compliance of analyzed compounds with these rules. The abbreviation of properties is in agreement with those in Table 1. Table S2. Predicted pharmacokinetic properties of analyzed compounds grouped under absorption, distribution, metabolism, excretion, and toxicity. Table S3. The toxicity profile of compounds as predicted by ProTox-II web server. For each target we used “I” for inactive and “A” for active. The probability is written in brackets. Figure S1. The binding probability of compounds to 18 crystal structures belonging to the following nuclear receptors: AR—androgen receptor, ERα—estrogen receptor alpha, ERβ—estrogen receptor beta, GR—glucocorticoid receptor, LXRα—liver X receptor alpha, LXRβ—liver X receptor beta, MR—mineralocorticoid receptor, PPARα—peroxisome proliferator-activated receptor alpha, PPARβ—peroxisome proliferator-activated receptor beta, PPARγ—peroxisome proliferator-activated receptor gamma, PR—progesterone receptor, RXRα—retinoid X receptor alpha, TRα—thyroid hormone receptor alpha, TRβ—thyroid hormone receptor beta. The high binding probability is marked with red (sensitivity < 0.25), orange (0.25 < sensitivity < 0.5) and yellow (0.5 < sensitivity < 0.75) were used to highlight decreasing probabilities, and green (sensitivity > 0.75) is used to mark the low binding probability. The docking scores are labeled next to each receptor, more negative values being associated with a higher affinity binding. Results were generated by the Endocrine Disruptome web server [20]. Figure S2. The structures of D2 and D3 compounds are colored according to the contributions of their atoms or fragments to hERG blockade. The fragments with a positive contribution are colored with green. Higher positive contributions are shown with a more intense green color and with more contour lines. The negative contributions to the blockade are highlighted in pink. Images were generated by Pred-hERG4.2 web server. Table S4. Molinspiration bioactivity scores v2021.03.
