# Supplementary material for: Biomedical Promise of Sustainable Microwave-Engineered Symmetric Curcumin Derivatives
Source: Pharmaceutics. 2024 Jan 31;16(2):205. doi: 10.3390/pharmaceutics16020205 (PMC10892556; doi:10.3390/pharmaceutics16020205)
Supplement: Supplementary file 1 [file pharmaceutics-16-00205-s001.zip › pharmaceutics-2814056-supplementary.pdf]

# Supplementary materials

## Biomedical Promise of Sustainable Microwave-Engineered Symmetric Curcumin Derivatives

**Cristina Doina Niţu, Maria Mernea, Raluca-Ioana Vlasceanu, Bianca Voicu-Balasea, Madalina Andreea Badea, Florentina Monica Raduly, Valentin Rădiţoiu, Alina Rădiţoiu, Speranta Avram, Dan F. Mihailescu, Ionela C. Voinea and Miruna Silvia Stan**

Table of contents:


- Table S1
- Table S2
- Table S3
- Figure S1
- Figure S2
- Table S4


**Table S1.** The acceptable values of compounds' physicochemical properties according to the drug-likeness rules of Lipinski, Ghose, Veber, Egan and Muegge , and the compliance of analyzed compounds with these rules. The abbreviation of properties is in agreement with those in Table 1.

| Rule | Acceptable ranges for compounds' properties | D1 enol | D1 keto | D2 enol | D2 keto |
|---|---|---|---|---|---|
| Lipinski | MW ≤ 500; MLPGP ≤ 4.15; HBA ≤ 10; HBD ≤ 5 | yes | yes | yes | yes |
| Ghose | 160 ≤ MW ≤ 480; -4 ≤ WLOGP ≤ 5.6; | yes | yes | yes | yes |
| Veber | 40 ≤ MR ≤ 130; 20 ≤ atoms ≤ 70 | yes | yes | yes | yes |
| Egan | NRB ≤ 10; TPSA ≤ 140 | yes | yes | yes | yes |
| Muegge | WLOGP ≤ 5.88; TPSA ≤ 131.6 | yes | yes | yes | yes |

**Table S2.** Predicted pharmacokinetic properties of analyzed compounds grouped under absorption, distribution, metabolism, excretion, and toxicity.

| | Property | D1 enol | D1 keto | D2 enol | D2 keto |
|---|---|---|---|---|---|
| **Absorption** | CaCo2 permeability (log Papp in $10^{-6}$ cm/s) | 0.598 | 0.382 | 1.106 | 1.02 |
| | Intestinal absorption human (% absorbed) | 78.154 | 83.25 | 89.571 | 90.805 |

| | | | | | |
|---|---|---|---|---|---|
| Distribution | Steady state volume of distribution -VDss (log L/kg) | -0.139 | -0.285 | -0.09 | -0.247 |
| | BBB permeability (log BB) | -0.772 | -0.19 | -0.679 | -0.207 |
| | CNS permeability (log PS) | -2.459 | -2.414 | -2.425 | -2.332 |
| Metabolism | CYP2D6 substrate (Yes/No) | No | No | No | No |
| | CYP3A4 substrate (Yes/No) | Yes | Yes | No | Yes |
| | CYP1A2 inhibitor (Yes/No) | No | No | Yes | Yes |
| | CYP2C19 inhibitor (Yes/No) | Yes | Yes | Yes | Yes |
| | CYP2C9 inhibitor (Yes/No) | Yes | Yes | Yes | Yes |
| | CYP2D6 inhibitor (Yes/No) | No | No | No | No |
| | CYP3A4 inhibitor (Yes/No) | Yes | Yes | Yes | Yes |
| Excretion | Total clearance (log ml/min/kg) | 0.36 | 0.199 | 0.205 | 0.041 |
| | Renal OCT2 substrate (Yes/No) | No | No | No | No |

**Table S3.** The toxicity profile of compounds as predicted by ProTox-II web server. For each target we used "I" for inactive and "A" for active. The probability is written in brackets.

| Classification | Target | Prediction (Probability) | | | |
|---|---|---|---|---|---|
| | | D1 enol | D1 keto | D2 enol | D2 keto |
| Organ toxicity | Hepatotoxicity | I (0.75) | I (0.60) | I (0.63) | I (0.65) |
| Toxicity endpoints | Carcinogenicity | I (0.94) | I (0.65) | I (0.69) | I (0.64) |
| | Immunotoxicity | I (0.98) | I (0.97) | I (0.96) | I (0.90) |
| | Mutagenicity | I (0.88) | I (0.74) | I (0.81) | I (0.82) |
| | Cytotoxicity | I (0.80) | I (0.71) | I (0.88) | I (0.93) |
| Tox21-Nuclear receptor signaling pathways | Aryl hydrocarbon Receptor (AhR) | I (0.98) | I (0.65) | I (0.74) | I (0.62) |
| | Androgen Receptor (AR) | I (0.88) | I (0.94) | I (0.82) | I (0.86) |
| | Androgen Receptor Ligand Binding Domain (AR-LBD) | I (0.86) | I (0.97) | I (0.99) | I (0.99) |
| | Aromatase | I (0.86) | I (0.88) | I (0.84) | I (0.86) |
| | Estrogen Receptor Alpha (ER) | I (0.67) | I (0.67) | A (0.67) | A (0.70) |
| | Estrogen Receptor Ligand Binding Domain (ER-LBD) | I (0.84) | I (0.97) | I (0.51) | I (0.52) |
| | Peroxisome Proliferator Activated Receptor Gamma (PPAR-Gamma) | I (0.92) | I (0.92) | I (0.78) | I (0.78) |

| | | | | | |
|---|---|---|---|---|---|
| Tox21-Stress response pathways | Nuclear factor (erythroid-derived 2)-like 2/antioxidant responsive element (nrf2/ARE) | I (0.75) | I (0.84) | I (0.73) | I (0.71) |
| | Heat shock factor response element (HSE) | I (0.94) | I (0.84) | I (0.73) | I (0.71) |
| | Mitochondrial Membrane Potential (MMP) | I (0.98) | I (0.58) | A (0.62) | A (0.78) |
| | Phosphoprotein (Tumor Suppressor) p53 | I (0.88) | I (0.81) | I (0.65) | I (0.66) |
| | ATPase family AAA domain-containing protein 5 (ATAD5) | I (0.80) | I (0.88) | I (0.75) | I (0.66) |

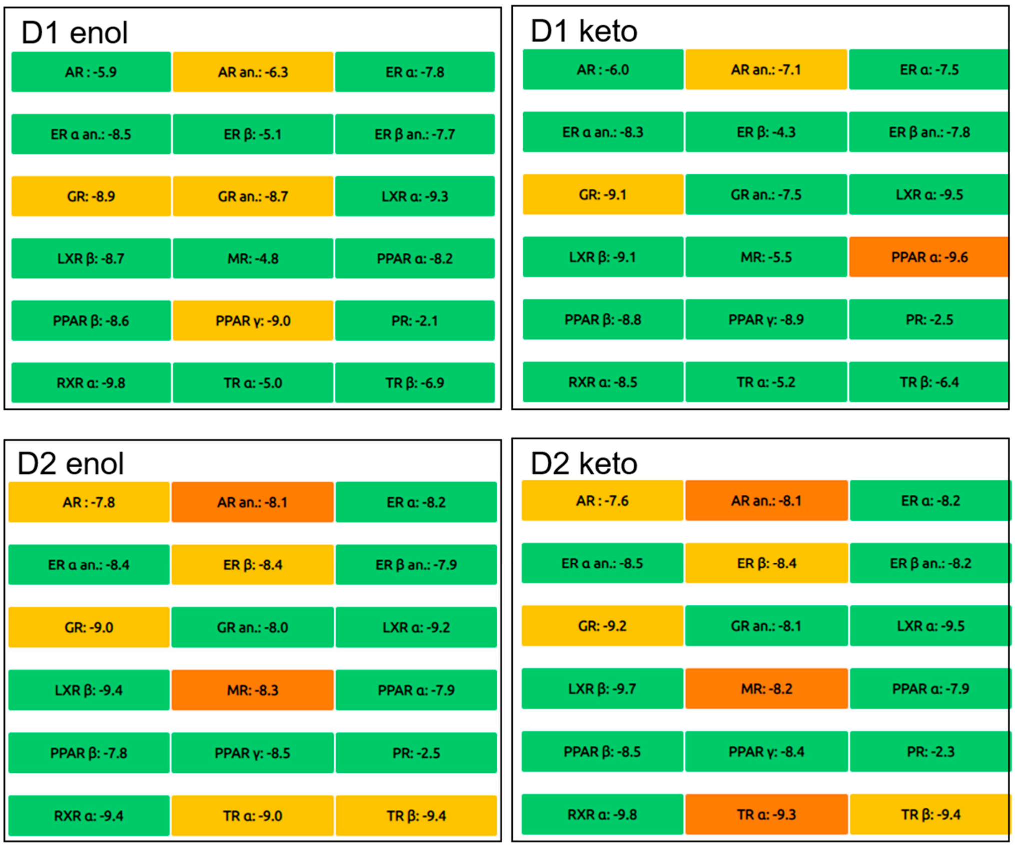


**Figure S1.** The binding probability of compounds to 18 crystal structures belonging to the following nuclear receptors: AR – androgen receptor, ER*α* – estrogen receptor alpha, ERβ – estrogen receptor beta, GR – glucocorticoid receptor, LXR*α* – liver X receptor alpha, LXRβ – liver X receptor beta, MR – mineralocorticoid receptor, PPAR*α* – peroxisome proliferator-activated receptor alpha, PPARβ – peroxisome proliferator-activated receptor beta, PPAR*γ* – peroxisome proliferator-activated receptor gamma, PR – progesterone receptor, RXR*α* – retinoid X receptor alpha, TR*α* – thyroid hormone receptor

alpha, TRβ – thyroid hormone receptor beta. The high binding probability is marked with red (sensitivity < 0.25), orange (0.25 < sensitivity < 0.5) and yellow (0.5 < sensitivity < 0.75) were used to highlight decreasing probabilities, and green (sensitivity > 0.75) is used to mark the low binding probability. The docking scores are labeled next to each receptor, more negative values being associated with a higher affinity binding. Results were generated by the Endocrine Disruptome web server [20].

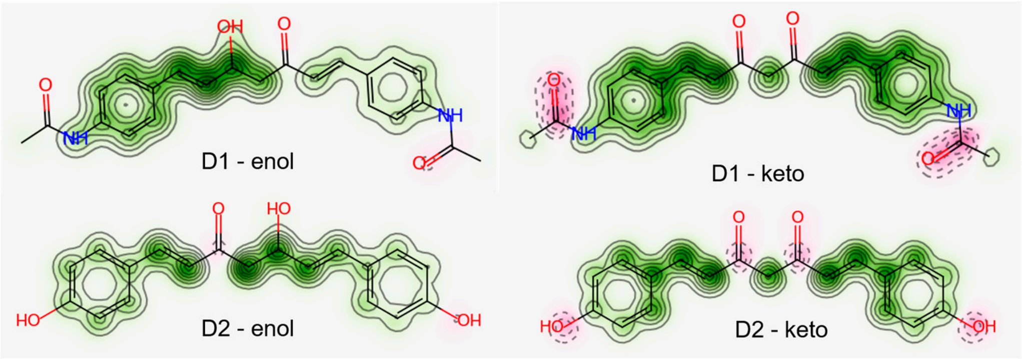


**Figure S2.** The structures of D2 and D3 compounds are colored according to the contributions of their atoms or fragments to hERG blockade. The fragments with a positive contribution are colored with green. Higher positive contributions are shown with a more intense green color and with more contour lines. The negative contributions to the blockade are highlighted in pink. Images were generated by Pred-hERG4.2 web server.

**Table S4.** Molinspiration bioactivity scores v2021.03.

| **Bioactivity** | **Predicted scores** | | | |
|---|---|---|---|---|
| | D1 enol | D1 keto | D2 enol | D2 keto |
| G-protein coupled receptor (GPCR) ligand | 0.04 | -0.10 | 0.17 | 0.00 |
| Ion channel modulator | -0.17 | -0.25 | -0.05 | -0.14 |
| Kinase inhibitor | -0.27 | -0.28 | -0.26 | -0.26 |
| Nuclear receptor ligand | -0.06 | -0.07 | 0.24 | 0.25 |
| Protease inhibitor | 0.04 | -0.11 | 0.08 | -0.08 |
| Enzyme inhibitor | 0.23 | -0.02 | 0.45 | 0.15 |
